# Uniform magnetic force impact on water based nanofluid thermal behavior in a porous enclosure with ellipse shaped obstacle

**DOI:** 10.1038/s41598-018-37964-y

**Published:** 2019-02-04

**Authors:** M. Sheikholeslami, Zahir Shah, Ahmad Shafee, Ilyas Khan, Iskander Tlili

**Affiliations:** 1 Department of Mechanical Engineering, Babol Noshirvani University of Technology, Babol, Iran; 2 Renewable energy systems and nanofluid applications in heat transfer Laboratory, Babol Noshirvani University of Technology, Babol, Iran; 3 Department of Mathematics, Abdul Wali Khan University, Mardan, KP Pakistan; 4 FAST, University Tun Hussein Onn Malaysia, 86400, Parit Raja, Batu Pahat, Johor State Malaysia; 5 Public Authority of Applied Education and Training, College of Technological Studies, Applied Science Department, Shuwaikh, Kuwait; 6 Faculty of Mathematics and Statistics, Ton Duc Thang University, Ho Chi Minh City, Vietnam; 7 Energy and Thermal Systems Laboratory, National Engineering School of Monastir, Street Ibn El Jazzar, 5019 Monastir, Tunisia

## Abstract

In the present research, aluminum oxide- water (Al_2_O_3_-H_2_O) nanofluid free convection due to magnetic forces through a permeable cubic domain with ellipse shaped obstacle has been reported. Lattice Boltzmann approach is involved to depict the impacts of magnetic, buoyancy forces and permeability on nanoparticles migration. To predict properties of Al_2_O_3_- water nanofluid, Brownian motion impact has been involved. Outcomes revels that considering higher magnetic forces results in greater conduction mechanism. Permeability can enhance the temperature gradient.

## Introduction

By suggesting nanoparticles from nanoscience as useful working fluid, thermal performance enhances. Nano sized metallic particles are dispersed into common fluid to generate such fluid. Nanofluids must be utilized to augment the conduction and can be more stable with better mixing^1,2^. Nano science can suggest appropriate working fluid to reach thermal efficiency enhancement^3–6^. The furthermost current publications on nanofluids with new applications can be demonstrated in^7–12^. Kumar *et al*.^13^ involved the Brownian motion impact on characteristics of nanoparticles in bioconvective flow. Irfan *et al*.^14^ displayed the roles of chemical terms on transient energy equation. Ahmed *et al*.^15^ illustrated the carbon nanotubes flow between Riga sheets in existence of viscous dissipation. Kumar *et al*.^16^ employed the non-Fourier heat flux model for investigation of magnetic force effect on Carreau fluid convective transient flow. Ali *et al*.^17^ demonstrated hidden events during magnetohydrodynamic (MHD) migration in a permeable media. Soomro *et al*.^18^ employed Finite difference method (FDM) for dual solution of nanoparticle migration over a cylinder. They used water as pure fluid. Reddy *et al*.^19^ depicted the impact of magnetic terms on fluid flow along a sheet considering heat sink. Raizah *et al*.^20^ illustrated the power law nanofluid natural convection inside a titled permeable duct. The furthermost recent articles about Nano sized particles transportation by involving various methods were reported by Shah *et al*.^9,21,22^. Choosing active working fluid becomes popular subject in recent decade^23–51^.

The main aim of current research is to simulate and examine nanoparticles migration within a cubic porous cavity under the influence of constant magnetic force. Hydrothermal behaviors for various permeability, Lorentz and buoyancy forces are mainly focused and shown through graph.

## Geometry Explanation

**Figure 1:**
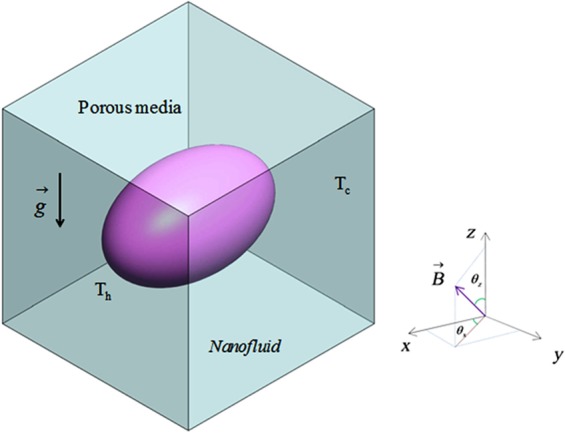
Current porous cubic cavity.

Figure 1 displays the permeable cubic cavity which is full of alumina. Cold, adiabatic and hot surfaces are depicted in this graph. One direction magnetic force has been involved. (*θ*_*z*_ = 0.5 *π* = *θ*_*x*_).

## Simulation by Mesoscopic Method

### Mesoscopic method

To find the temperature and velocity, distribution functions were used namely (g and f). Boltzmann equations help to find functions *g* and *f*. According to assumptions exist in^38^, we have:

1 $${\rm{\Delta }}t\,{\tau }_{C}^{-1}[\,-\,{g}_{i}(x,t)+{g}_{i}^{eq}(x,t)]={g}_{i}(x+{\rm{\Delta }}t\,{c}_{i},t+{\rm{\Delta }}t)-{g}_{i}(x,t)$$

2 $${\rm{\Delta }}t\,{\tau }_{v}^{-1}[\,-\,{f}_{i}(x,t)+{f}_{i}^{eq}(x,t)]+{f}_{i}(x,t)+{\rm{\Delta }}t{c}_{i}{F}_{k}={f}_{i}(x+{\rm{\Delta }}t\,{c}_{i},t+{\rm{\Delta }}t)$$

Here *τ*_*c*_, Δ*t*, *τ*_*v*_ and *c*_*i*_ are, relaxation time for *T*, time step, relaxation time for *u* and lattice velocity.

**Figure 2:**
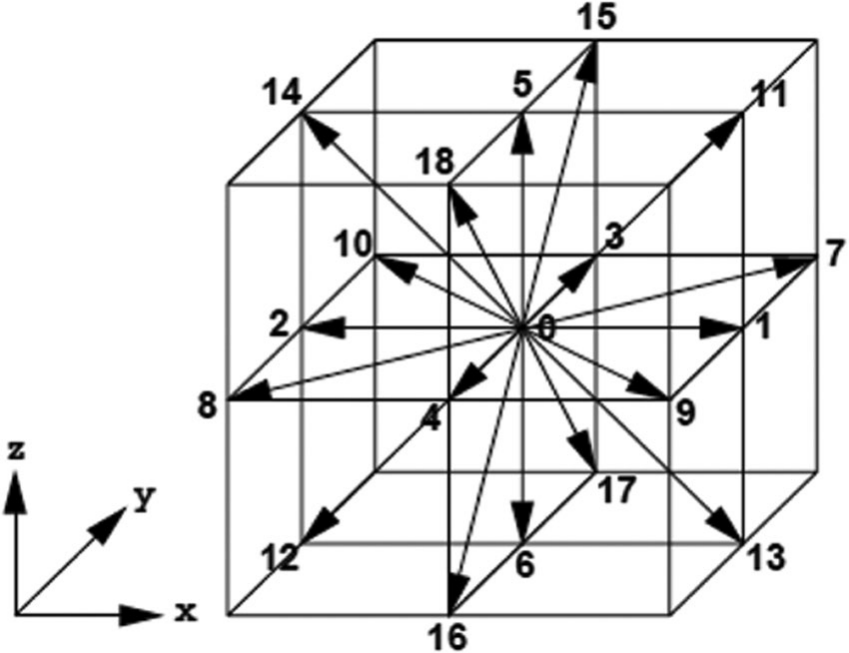
Diagram of D_3_Q_19_ model.

D_3_Q_19_ model is good method for such problem (as shown in Fig. 2):

3 $${c}_{i}=(\begin{array}{llllllllllllllllll}0 & 0 & 0 & 0 & -1 & -1 & -1 & -1 & 1 & 1 & 1 & 0 & 0 & 0 & 0 & -1 & 1 & 0\\ -1 & -1 & 1 & 1 & 0 & -1 & -1 & 1 & 0 & -1 & 1 & 0 & 0 & -1 & 1 & 0 & 0 & 0\\ -1 & 1 & -1 & 1 & -1 & 0 & 0 & 0 & 1 & 0 & 0 & -1 & 1 & 0 & 0 & 0 & 0 & 0\end{array})$$

$${g}_{i}^{eq}$$ & $${f}_{i}^{eq}$$ are:

4 $${g}_{i}^{eq}={w}_{i}T[1+\frac{{c}_{i}.u}{{c}_{s}^{2}}]$$

5 $${f}_{i}^{eq}=[\frac{1}{2}\frac{{({c}_{i}.u)}^{2}}{{c}_{s}^{4}}-\frac{1}{2}\frac{{u}^{2}}{{c}_{s}^{2}}+1+\frac{{c}_{i}.u}{{c}_{s}^{2}}]{w}_{i}\rho $$

6 $${w}_{i}=\{i=7:18\,\,1/36;\,\,i=0\,\,1/3;\,\,i=1:6\,\,1/18\}$$

Body forces can calculate as:

7 $$\begin{array}{rcl}F & = & {F}_{y}+{F}_{z}+{F}_{x}\\ {F}_{y} & = & \begin{array}{c}3A{w}_{i}\rho [-(\,-\,\sin (2{\theta }_{z})\sin ({\theta }_{x})(0.5)w+v{\cos }^{2}({\theta }_{z}))\\ +(-v{\cos }^{2}({\theta }_{x}){\sin }^{2}({\theta }_{z})+0.5u\,\sin (2{\theta }_{x}){\sin }^{2}({\theta }_{z}))]-3BB\,{w}_{i}\rho v,\end{array}\\ {F}_{x} & = & 3{w}_{i}A\rho [-\sin ({\theta }_{x})(-v\,{\sin }^{2}({\theta }_{z})\cos ({\theta }_{x})+\,\sin ({\theta }_{x})u\,{\sin }^{2}({\theta }_{z}))\\  &  & -(u\,{\cos }^{2}({\theta }_{z})+w\,\cos ({\theta }_{x})(\frac{1}{2})\sin (2{\theta }_{z}))]-BB(3)\rho {w}_{i}u,\\ {F}_{z} & = & 3{w}_{i}\rho [A\,\cos ({\theta }_{x})(-\cos ({\theta }_{x}){\sin }^{2}({\theta }_{z})w+(\frac{u}{2})\cos (2{\theta }_{z}))\\  &  & +\,\sin ({\theta }_{x})A(-\sin ({\theta }_{x}){\sin }^{2}({\theta }_{z})w+\,\sin (2{\theta }_{z})\frac{v}{2})+\beta (T-{T}_{m}){g}_{z}]-BB(3{w}_{i})w\rho ,\\ Ha & = & L{B}_{0}\sqrt{\frac{\sigma }{\mu }},\,A=H{a}^{2}\mu {L}^{-2},\\ Da & = & \frac{K}{{L}^{2}},\,BB=\frac{\upsilon }{Da\,{L}^{2}}\end{array}$$

To calculate scholars we have:

8 $$\begin{array}{c}{\rm{Flow}}\,{\rm{density}}:\rho =\sum _{i}\,{f}_{i},\\ {\rm{Momentum}}:\rho {\rm{u}}=\sum _{i}{{\rm{c}}}_{i}\,{f}_{i},\\ {\rm{Temperature}}:T=\sum _{i}\,{g}_{i}.\end{array}$$

### Working fluid

Density, (*ρβ*)_*nf*_, (*ρC*_*p*_)_*nf*_, *σ*_*nf*_, *μ*_*nf*_ and *k*_*nf*_ are (^39^):

9 $$\frac{{\rho }_{nf}}{{\rho }_{f}}=-\,\varphi +\frac{{\rho }_{s}}{{\rho }_{f}}\varphi +1,$$

10 $${(\rho \beta )}_{nf}=\varphi {(\rho \beta )}_{s}+(1-\varphi ){(\rho \beta )}_{f}$$

11 $${(\rho {C}_{p})}_{nf}/{(\rho {C}_{p})}_{f}=-\,\varphi +1+{(\rho {C}_{p})}_{s}/{(\rho {C}_{p})}_{f}\varphi $$

12 $$\frac{{\sigma }_{nf}}{{\sigma }_{f}}=1+{(\frac{({\rm{\Delta }}+2)-\varphi ({\rm{\Delta }}-1)}{3\varphi (-1+{\rm{\Delta }})})}^{-1},\,{\rm{\Delta }}={\sigma }_{s}/{\sigma }_{f}$$

13 $${\mu }_{nf}=\frac{{\mu }_{f}}{{(1-\varphi )}^{2.5}}+\frac{{\mu }_{f}}{\Pr }\frac{{k}_{Brownian}}{{k}_{f}}$$

14 $$\begin{array}{c}\frac{{k}_{nf}}{{k}_{f}}=1+5\times {10}^{4}g^{\prime} (\varphi ,T,{d}_{p})\varphi {\rho }_{f}{c}_{p,f}\sqrt{\frac{{\kappa }_{b}T}{{d}_{p}{\rho }_{p}}}+\frac{3(-1+\frac{{k}_{p}}{{k}_{f}})\varphi }{(\frac{{k}_{p}}{{k}_{f}}+2)-(\frac{{k}_{p}}{{k}_{f}}-1)\varphi },\\ {R}_{f}=4\times {10}^{-8}k{m}^{2}/W,\,{R}_{f}=-{d}_{p}(1/{k}_{p}-1/{k}_{p,eff}),\\ g^{\prime} (\varphi ,{d}_{p},T)=Ln(T)({a}_{1}+{a}_{3}Ln(\varphi )+Ln{({d}_{p})}^{2}{a}_{5}+{a}_{2}Ln({d}_{p})+{a}_{4}Ln({d}_{p})Ln(\varphi ))\\ +({a}_{8}Ln(\varphi )+Ln({d}_{p}){a}_{7}+{a}_{6}+{a}_{10}Ln{({d}_{p})}^{2}+Ln({d}_{p}){a}_{9}Ln(\varphi ))\end{array}$$

Tables 1 and 2^39^ can be used to find needed parameters. *Nu*_*ave*_ and *Nu*_*loc*_ over the hot surface are:

15 $$N{u}_{loc}=-\frac{{k}_{nf}}{{k}_{f}}{\frac{\partial T}{\partial X}|}_{X=0}\,{\rm{and}}\,N{u}_{ave}={\int }_{0}^{1}{\int }_{0}^{1}Nu\,dYdZ$$

**Table 1:** Properties of *Water*, *Al*_2_*O*_3_.

	σ(Ω · m)^−1^	k(W/m.k)	C_p_(j/kgk)	ρ(kg/m^3^)
Pure water	0.05	0.613	4179	997.1
Al_2_O_3_	10^−12^	25	765	3970

**Table 2:** Related coefficient for alumina.

Coefficient values	Al_2_O_3_−Water
a_6_	−298.19819084
a_7_	−34.532716906
a_8_	−3.9225289283
a_9_	−0.2354329626
a_10_	−0.999063481
a_1_	52.813488759
a_2_	6.115637295
a_3_	0.6955745084
a_4_	4.174555527E-02

The fluid kinetic energy is:

16 $${E}_{c}=0.5[{(w)}^{2}+{(v)}^{2}+{(u)}^{2}]$$

## Mesh Independency and Validation

**Figure 3:**
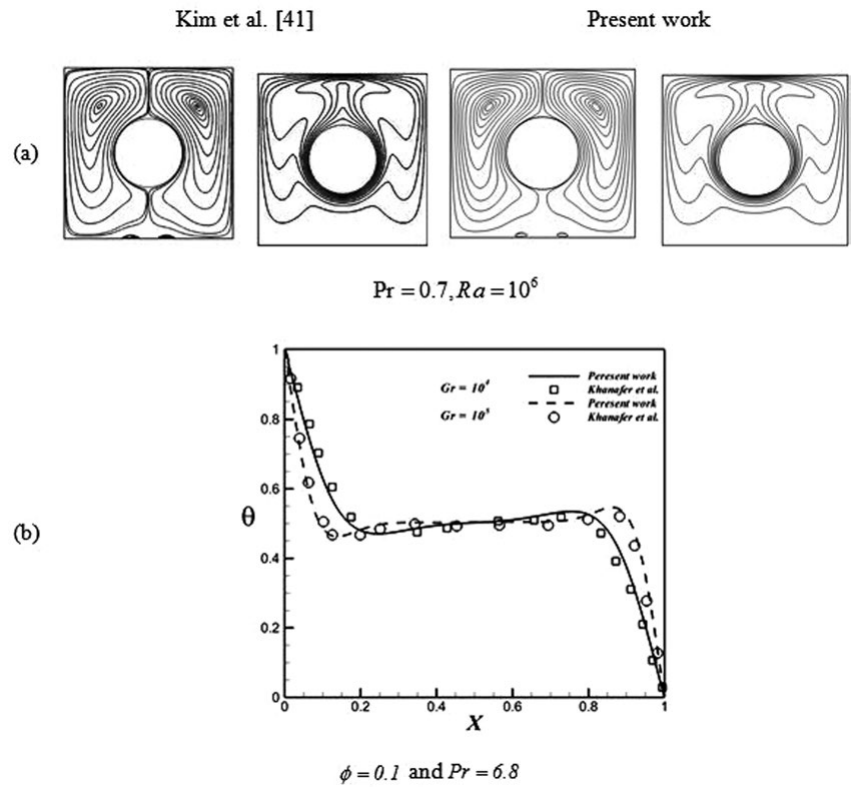
Verification of current LBM code for (**a**) free convention^40^; (**b**) nanofluid flow^41^.

No alter should be seen in outputs by changing mesh sizes. So, various sizes must be employed. As an example, we presented Table 3. Figure 3 illustrates the agreement of Lattice Boltzmann Method (LBM)^40,41^. Also, previous paper^42^ indicates that this code is verified for MHD flow.

**Table 3:** *Nu*_*ave*_ over the hot surface with various grid sixes when *Da* = 100, *ϕ* = 0.04, *Ra* = 10^5^, and *Ha* = 60.

Mesh size	51 × 51 × 51	61 × 61 × 61	71 × 71 × 71	81 × 81 × 81	91 × 91 × 91
Nu_ave_	0.13622	0.14805	0.15061	0.15073	0.15097

## Results and Discussion

Water-Aluminum oxide mixture hydrothermal behavior in a permeable three dimensional domain was modeled with mesoscopic method. Numerical outputs are depicted the variations of magnetic force (*Ha* = 0 to 60), buoyancy term ($Ra={10}^{3},\,{10}^{4}$ and 10^5^) and Darcy number (*Da* = 0.001 to 100).

**Figure 4:**
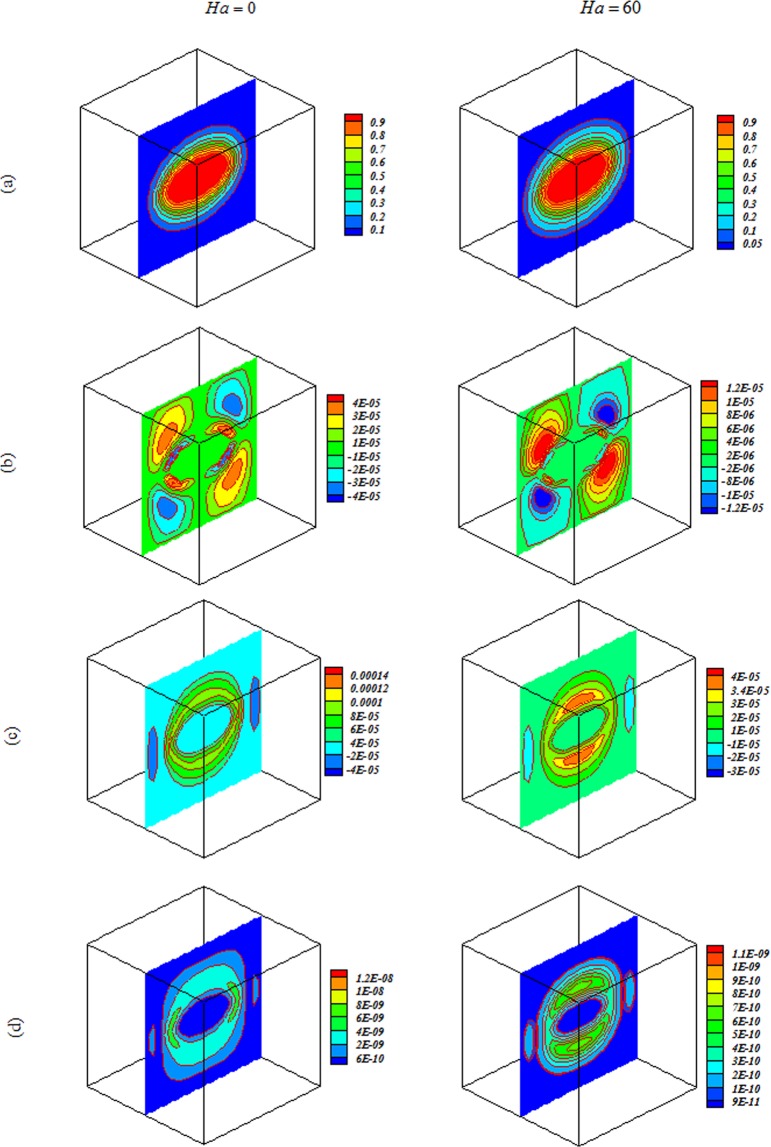
Impacts of magnetic forces on (**a**) isotherm, (**b**) *x* velocity, (c) *z* velocity, (**d**) isokinetic energy at *Y* = *y*/*L* = 0.5 when *ϕ* = 0.04, *Da* = 0.001, *Ra* = 10^3^.

**Figure 5:**
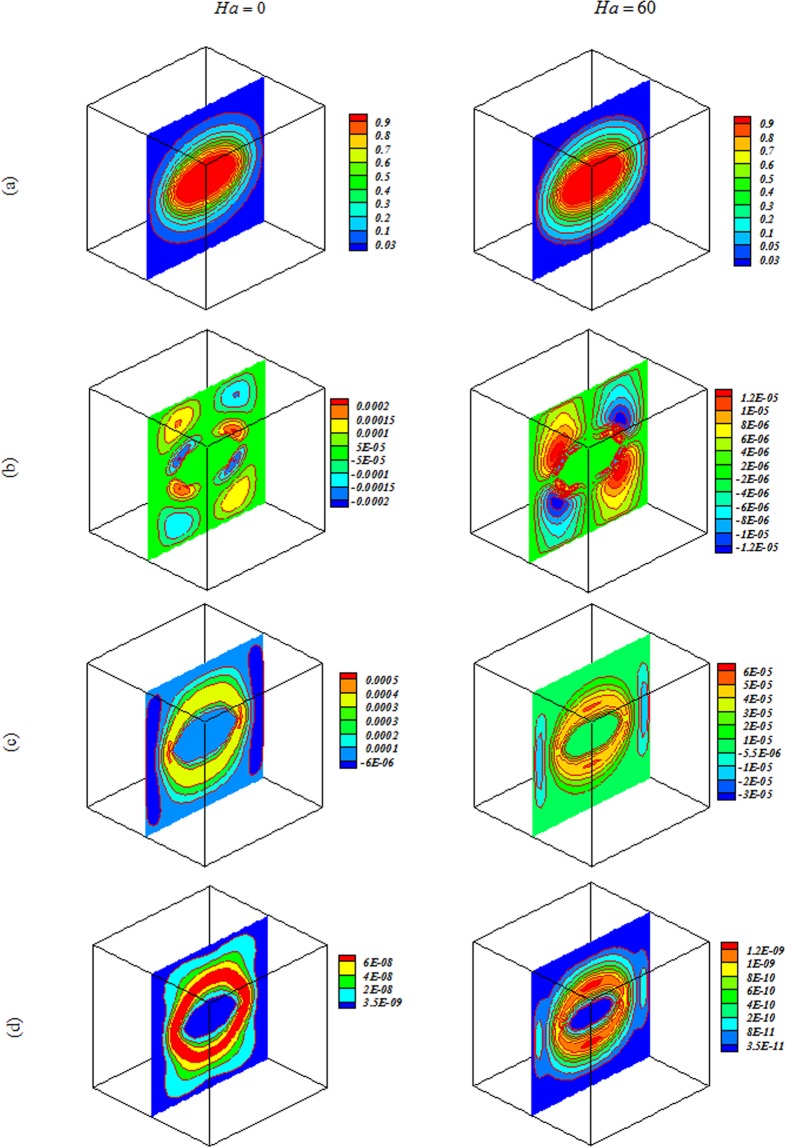
Impacts of magnetic forces on (**a**) isotherm, (**b**) *x* velocity, (**c**) *z* velocity, (**d**) isokinetic energy at *Y* = *y*/*L* = 0.5 when *Ra* = 10^3^, *Da* = 100, *ϕ* = 0.04.

**Figure 6:**
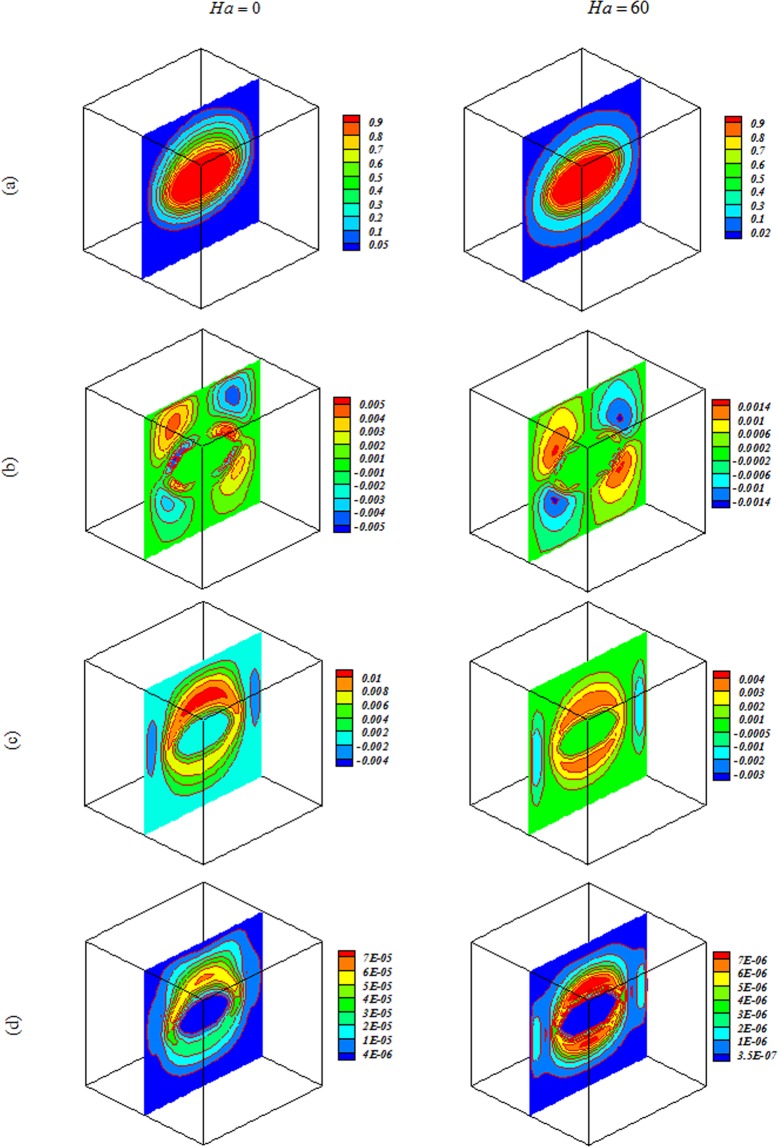
Impacts of magnetic forces on (**a**) isotherm, (**b**) *x* velocity, (**c**) *z* velocity, (**d**) isokinetic energy at *Y* = *y*/*L* = 0.5 when *Ra* = 10^5^, *Da* = 0.001, *ϕ* = 0.04.

**Figure 7:**
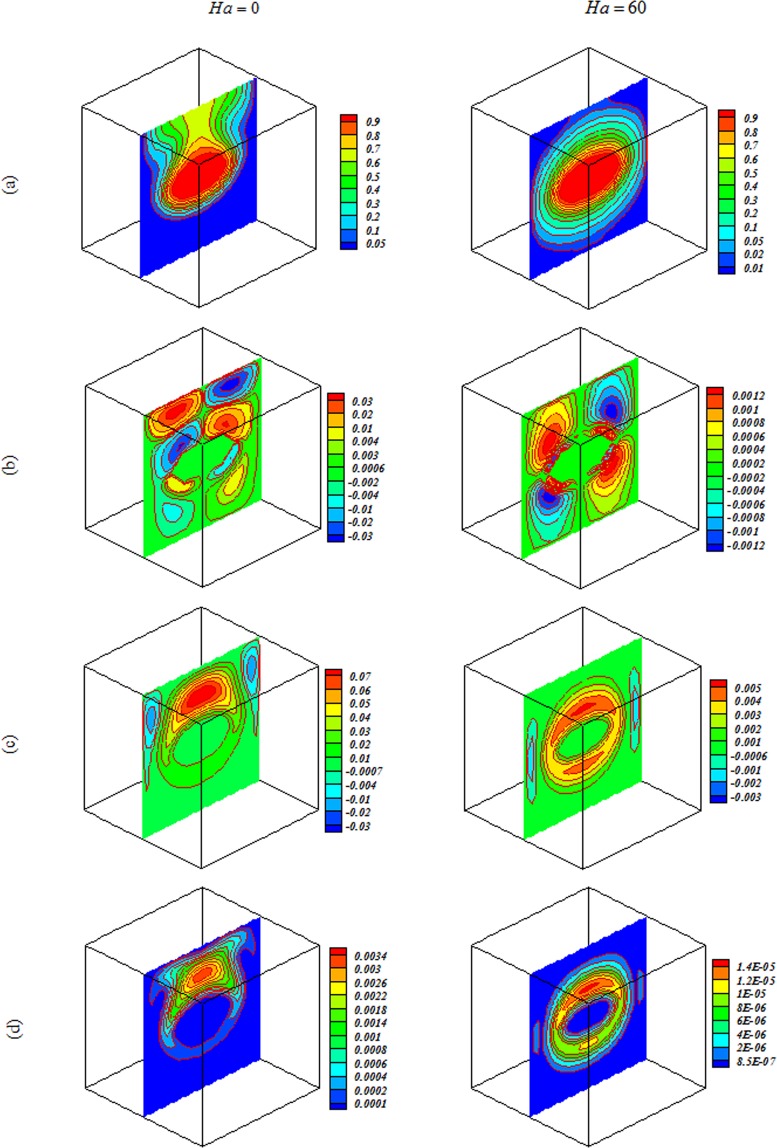
Impacts of magnetic forces on (**a**) isotherm, (**b**) *x* velocity, (**c**) *z* velocity, (**d**) isokinetic energy at *Y* = *y*/*L* = 0.5 when *Ra* = 10^5^, *Da* = 100, *ϕ* = 0.04.

Nanofluid behavior with change of $Ra,Ha$ and *Da* are displayed in Figs 4–7. In cases with low $Ra$ and *Da*, convection mode is not strong enough to change flow style and isotherms has shape of geometry. Convection enhancements with increase of permeability and isotherms convert to complex shape. Thermal plume appears as a result of strong convection mode. Employing magnetic forces makes conduction to be more sensible and thermal plumes vanish. Due to reduction effect of Ha on velocity, *E*_*c*_ detracts with rise of Ha. By augment of buoyancy force, main vortex stretch in z direction and convection mode rises.

**Figure 8:**
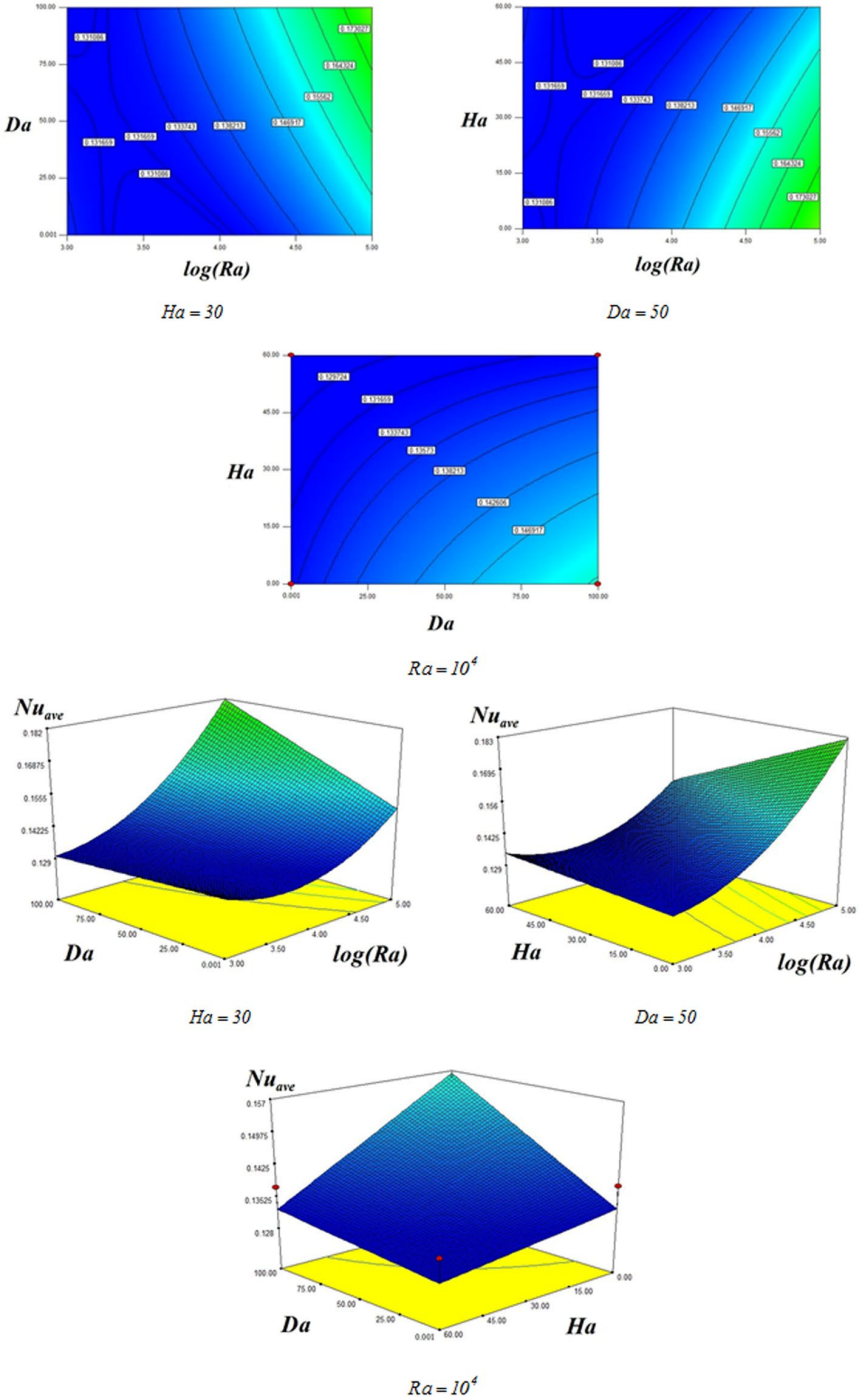
Various values of *Nu*_*ave*_ for different *Ra*, *Da*, *Ha*.

Changes in *Nu*_*ave*_ due to altering variables are illustrated in Fig. 8. Equation (17) is extracted for *Nu*_*ave*_:

17 $$N{u}_{ave}=0.14+0.017\,\mathrm{log}(Ra)+6.8\times {10}^{-3}Da-7.2\times {10}^{-3}Ha+9\times {10}^{-3}(\mathrm{log}(Ra))(Da)-9\times {10}^{-3}(\mathrm{log}(Ra))(Ha)-4.7\times {10}^{-3}(Da)(Ha)+0.012{(\mathrm{log}(Ra))}^{2}.$$

Due to augment in temperature gradient with rise of permeability and buoyancy terms, *Nu*_*ave*_ is enhancing function of $Da,Ra$. Furthermore, conduction mode boosts with augment of Hartmann number. Thus, *Nu*_*ave*_ detracts with rise of magnetic force.

## Conclusions

In the current article, uniform magnetic force impacts on momentum equations were considered in a 3D porous enclosure. Mesoscopic approach was applied to analyze alumina nanofluid in these conditions. Brownian motion impact can changes the properties of working fluid. LBM was involved to report the impacts of *Ha*, *Ra, Da* on nanofluid behavior. Outcomes display that interaction of nanoparticles augments with augment of *Da*,*Ra*. Isotherms become less complex with applying magnetic force.
